# Emerging agents and regimens for hepatocellular carcinoma

**DOI:** 10.1186/s13045-019-0794-6

**Published:** 2019-10-26

**Authors:** Xiao-Dong Zhu, Hui-Chuan Sun

**Affiliations:** Department of Liver Surgery and Transplantation, Liver Cancer Institute and Zhongshan Hospital, Fudan University, Shanghai, 200032 China

**Keywords:** Hepatocellular carcinoma, Systemic therapy, Molecular targeted therapy, Anti-PD-1 antibody

## Abstract

Liver cancer, mostly hepatocellular carcinoma (HCC), is the second leading cause of cancer mortality globally. Most patients need at least one systemic therapy at different phases of their treatment for HCC. Sorafenib was the first agent shown to improve the survival of patients with advanced HCC. A decade after the approval of sorafenib, most agents failed to improve patient survival more than sorafenib. In recent years, treatment practices have changed, with lenvatinib as another first-line treatment choice and regorafenib, ramucirumab, and cabozantinib as second-line treatment options. Anti-PD-1 antibodies, including nivolumab, pembrolizumab, and camrelizumab, have demonstrated promising anti-tumor effects as monotherapy for advanced HCC in phase II clinical trials. The combination of an anti-PD-1 antibody and an anti-angiogenesis agent has shown more potent anti-tumor effects in early phase clinical trials and is now the hotspot in clinical studies. Furthermore, these agents are investigated in combination treatment with surgery or other loco-regional therapies in patients with early or intermediate-stage HCC.

## Background

Primary liver cancer is the second leading cancer-related death globally and ranks second in cancer mortality in China [1]. Although the incidence and mortality of liver cancer in China is declining [2, 3], largely owing to the introduction of vaccination for newborns against the hepatitis B virus [4], it is increasing in the USA and Europe [5]. More than 90% of primary liver cancers are hepatocellular carcinoma (HCC), and around 5–10% of primary liver cancers are intrahepatic cholangiocarcinoma. Curative treatment to provide long-term survival for patients with early stage HCC includes surgical resection, radiofrequency ablation, or liver transplantation. Transcatheter chemoembolization (TACE) is the standard treatment for patients with intermediate stage HCC [6]. The effect of systemic treatment for advanced stage liver cancer was disappointing until the approval of sorafenib in 2008.

The survival of HCC patients is poorer than many other types of cancer. In China, the 5-year survival of HCC is 12.1%, the second lowest among all types of cancer [7]. In most patients, HCC is associated with chronic liver injuries from hepatitis virus infection, alcohol abuse or non-alcoholic liver steatosis hepatitis, which not only complicates treatment choice, but also competes the effect of tumor progression on patient survival [8]. The treatment toxicities in liver cancer patients usually out-weight that in other cancers.

For patients with early stage HCC, surgical treatment, ablation or liver transplantation, may provide longer survival; however, they are associated with a high risk of tumor recurrence and no adjuvant treatment is accepted as a standard care [9]. In China, most HCC patients are diagnosed at advanced stages [10], and systemic treatment is the only option to improve survival.

## Approved agents for HCC

### Sorafenib: the only approved systemic therapy for a decade

Sorafenib has been approved for the treatment of advanced HCC for more than 10 years. Two trials conducted within and outside Asia have shown the efficacy of sorafenib in extending patient survival [11, 12]. Sorafenib became a standard of care recommended by the guidelines from almost all regions, and management of its toxicities, such as hand-foot syndrome, has improved its tolerance [13]. It has been estimated that the survival of patients with advanced stage HCC has been extended from 6.5 months to 8.5–8.9 months in Asian patients and from 10.7 months to 11.8–15.1 months in non-Asian patients, probably because of the improved management of toxicities associated with sorafenib treatment [14]. Attempts to identify a molecular biomarker for the selection of patients sensitive to sorafenib has, however, failed, although several reports demonstrated toxicities associated with better tumor response. Monotherapy with sunitinib [15], brivanib (BRISK-FL study [16]), linifanib [17], or selective internal radiotherapy with yttrium-90 resin microspheres (SARAH and SIRveNIB studies [18, 19]) had been shown not to be superior to sorafenib in head-to-head phase III trials until the REFLECT trial [20] demonstrated that lenvatinib is not inferior to sorafenib in terms of patient survival, followed by administrative approval.

Sorafenib has also been tested in other scenarios. Combination treatment with TACE has been intensively investigated, although most failed to demonstrate the additional benefit of sorafenib over TACE, while one retrospective analysis showed sorafenib may improve the survival of patients who were concomitantly treated with TACE [21]. Recently, the results from the TACTICS trial demonstrated that TACE plus sorafenib is more effective in prolonging progression-free survival (PFS) than TACE alone in patients with unresectable HCC, but the overall survival (OS) was not reported [22]. A recent randomized control trial (RCT) demonstrated the effect of sorafenib and hepatic arterial infusion using oxaliplatin, 5-fluorouracil, and leucovorin is better than sorafenib alone in patients with tumor invasion to the portal vein in terms of OS and PFS [23]. The combination of sorafenib and erlotinib (SEARCH study [24]), TACE (STAH study [25]), doxorubicin (CALGB 80802 study [26]), or hepatic arterial infusion with low-dose cisplatin and fluorouracil (SILIUS study [27]) failed to reach the pre-designated objectives.

The STORM trial to evaluate the effect of adjuvant sorafenib treatment after resection or ablation of early stage HCC (BCLC stage 0-A) with a high risk of tumor recurrence did not reach the expected objective [28]. The 1-year and 2-year tumor recurrence rates in the control arm were around 30% and 40%, suggesting more than 60% of patients may be not the target population for receiving adjuvant anti-tumor treatment. “Wrong stage and wrong dose” were the major criticisms for this trial [29]. Several retrospective studies have shown that sorafenib is effective in inhibiting tumor recurrence after resection of HCC with a higher risk of tumor recurrence, where the risk was much higher than in the STORM trial [30, 31]. A small RCT showed that sorafenib improved patient OS and decreased tumor recurrence rate only in those with a higher risk of tumor recurrence [32]. Lately, the surgical samples from the STORM trial were analyzed to establish a link between treatment efficacy and molecular profiling, and the results showed no mutation, gene amplification, or previously proposed gene signatures predicted sorafenib benefit [33].

### Lenvatinib

Lenvatinib is a multi-kinase inhibitor targeting vascular endothelial growth factor receptors (VEGFRs) 1–3, fibroblast growth factor receptors (FGFR) 1–4, platelet-derived growth factor receptor (PDGFR) α, RET, and KIT [34]. Lenvatinib was approved for advanced HCC in 2018 based on a non-inferior designed open-labeled control trial [20]. Although there are some doubts concerning the trial design, lenvatinib has been accepted because of its higher objective response rate (ORR), which is 18.8% judged by Response Evaluation Criteria in Solid Tumors (RECIST) 1.1 or 40.6% by the modified RECIST (mRECIST) by masked independent image review [20]. A real-world study demonstrated that therapeutic response and adverse events after taking lenvatinib were similar with the REFLECT trial, regardless of past tyrosine-kinase inhibitor (TKI) therapies [35], and its immunomodulatory activity has also been revealed in both experimental study [36] and clinical study [37].

Although the trial demonstrated that lenvatinib provided a similar survival benefit with sorafenib, the higher tumor response rate is very important to encourage patients to stay on treatment and tolerate toxicities and for physicians to monitor the effect of treatment. The higher tumor response rate also inspired thought of down-staging treatment for initially unresectable HCC or neoadjuvant therapy for resectable HCC. Furthermore, the REFLECT trial showed lenvatinib may be more effective in hepatitis B virus-infected HCC patients [20], while sorafenib may be more effective in hepatitis C virus infected-HCC patients [38], although the mechanism has not been revealed yet.

There are still some concerns regarding lenvatinib, however. In the REFLECT trial [20], PFS gain in the lenvatinib-treated arm did not translate into OS benefit, and the reason for this is not clear. A post hoc study showed more patients from the sorafenib-treated group received the investigating drug and cabozantinib (9.5% vs 3.1%, 2.3% vs 0%, respectively) [39]. Although the NCCN guideline for the treatment of HCC recommends sorafenib as the second-line treatment for patients who failed lenvatinib, a controlled study is needed to verify efficacy and explore other treatment choices. Finally, a biomarker for the selection of patients who may benefit from lenvatinib has not yet been identified. One study demonstrated that the presence of adverse effect in patients receiving lenvatinib was associated with a better OS [40].

### Regorafenib

Regorafenib is also a multi-target TKI, targeting VEGFRs 1–3, Tie-2, PDGFR-β, FGFRs, Kit, and Ret. The RESORCE trial [41] was conducted in patients who tolerated sorafenib but progressed on sorafenib treatment. The OS in regorafenib-treated patients was 10.6 months compared to 7.8 months in the placebo-treated patients (HR = 0.61, *P* < 0.0001), and PFS increased from 1.5 months to 3.1 months by regorafenib treatment (HR = 0.46, *P* < 0.0001). Regorafenib is the first second-line treatment showing an OS benefit, and regorafenib is more potent than sorafenib in terms of tumor response. The incidence of treatment-related grade 3 or 4 adverse event was 50%, including hand-foot syndrome, infection, hypertension, and fatigue.

The introduction of regorafenib has fundamentally changed the clinical management of HCC. Progression on sorafenib treatment became a clear signal to switch to regorafenib treatment. One study showed sequential treatment using sorafenib and regorafenib may result in 28 months of OS in patients with advanced HCC [42].

### Cabozantinib

Cabozantinib is a multi-kinase inhibitor targeting VEGFR-2, MET, and AXL. A randomized control study demonstrated cabozantinib treatment resulted in a longer OS (10.2 vs 8.0 months, HR = 0.76, *P* = 0.005) and PFS (5.2 vs 1.9 months, HR = 0.44, *P* < 0.001) in patients with advanced HCC as a second-line treatment [43]. An interesting finding from this study was that the hazard ratio for death was 0.69 in patients with a disease caused by HBV and 1.11 in patients with HCV, which suggests that cabozantinib may be more potent for HBV-related HCC.

The molecular target of cabozantinib, MET and AXL, have a role in treatment resistance to anti-angiogenesis therapies, which is consistent with the effect of cabozantinib as a second-line treatment for HCC. Compared with regorafenib, cabozantinib resulted in longer PFS (5.2 vs 3.4 months, per RECIST 1.1 [41, 43]), while grade 3 and 4 adverse events were more common in cabozantinib-treated patients, including hypertension, diarrhea, and hand-foot syndrome.

### Ramucirumab

Ramucirumab is an antibody targeting VEGFR-2. VEGFR-2 is the receptor on endothelial cells, whose ligands are VEGF-A, C, and D. Ramucirumab has been approved for the treatment of several other cancers, such as advanced gastric cancer, colorectal cancer, and non-small cell lung cancer. In the REACH trial in patients with advanced HCC (BCLC-B/C) who have been treated with sorafenib without success, prespecified subgroup analysis revealed that patients with AFP ≥ 400 ng/mL may benefit from ramucirumab treatment [44]. The REACH-2 trial was therefore conducted specifically in patients with AFP ≥ 400 ng/mL, and the results demonstrated that OS and PFS were significantly better than in the control arm [45].

The grade 3 or 4 adverse events associated with ramucirumab were very low. The median treatment intensity was 98% in the ramucirumab-treated group, suggesting that most patients received a full dose of ramucirumab, and adverse events leading to treatment discontinuation occurred in 11% of patients. Hypertension and hyponatremia were the only grade 3 or worse treatment-emergent adverse events that were noted in 5% or more of patients [45].

### PD-1/PD-L1 antibodies

Both nivolumab and pembrolizumab have been approved for the second-line treatment of advanced HCC by the USFDA, based on the results from two single-arm studies CheckMate 040 [46] and KEYNOTE-224 trials [47]. In the CheckMate 040 trial, nivolumab demonstrated an ORR for HCC of 20% as a first-line treatment or 14% as a second-line treatment (RECIST v1.1), and median OS (mOS) was 28.6 (95% CI, 16.6—not reached at data cutoff) months as a first-line treatment or 15.6 (95% CI, 13.0–18.9) months as a second-line treatment [48]. Similarly, the KEYNOTE-224 trial using pembrolizumab demonstrated an ORR of 17% (RECIST 1.1), and mOS was 12.9 months as a second-line treatment. Notably, the grade 3 or 4 treatment-related adverse effects were much lower than for TKIs, which were 19% in nivolumab-treated patients and 26% in pembrolizumab-treated patients as a second-line treatment, compared with 50% in regorafenib-treated patients and 68% in cabozantinib-treated patients [41, 43].

The KEYNOTE-240, a RCT to evaluate the efficacy of pembrolizumab as a second-line treatment, failed [49]. In this study, pembrolizumab did show a trend of better OS (HR = 0.78, 95% CI, 0.611–0.998, *P* = 0.0238) and PFS (HR = 0.78, 95% CI, 0.61–0.99, *P* = 0.0209) without statistical significance per the prespecified statistical plan. However, the magnitude of benefit as captured by HR for both primary endpoints and duration of response is consistent with the findings of KEYNOTE-224. It is noteworthy that more patients in the placebo arm received post-study anti-cancer therapy than those in the pembrolizumab-treated arm. The KEYNOTE-394, designed like KEYNOTE-240, is an ongoing trial in Asian patients with advanced HCC. Recently, Bristol-Myers Squibb announced the results of CheckMate-459, comparing nivolumab and sorafenib as first-line therapy for advanced HCC [50]. Although nivolumab monotherapy did show anti-tumor effects, the study did not achieve statistical significance for its primary endpoint of OS (HR = 0.85, 95% CI, 0.72–1.02, *P* = 0.0752).

The third PD-1 antibody agent that has been intensively evaluated in HCC is camrelizumab (SHR-1210, Hengrui Pharmaceutical, China). A phase II study demonstrated ORR as a second-line treatment was 13.8% (RECIST v1.1), and the mOS was estimated at 14.4 months (95% CI, 13.8—not reached at data cutoff). The grade 3 or 4 treatment-related adverse effect was 19.4% [51]. A unique adverse effect related with camrelizumab treatment is reactive capillary hemangioma [52], and a total of 66.8% of HCC patients who received camrelizumab monotherapy developed reactive capillary hemangioma [51]. The exact mechanism and its association with tumor response are not clear. However, the incidence of reactive capillary hemangioma was 20% when those patients were treated with a combination of camrelizumab and gemcitabine plus cisplatin [53], and 12.1% in patients treated by a combination of apatinib (a VEGFR-2 inhibitor) at a dose of 250 mg per day and camrelizumab [54].

Although the treatment-related adverse events of grade 3 or greater were relatively low for PD-1 antibodies compared with TKIs, early detection and management of these adverse events are even more important as some of them (e.g., myocarditis, pneumonitis, hepatitis, adrenal insufficiency, and myositis) may be fatal [55]. For patients with a large tumor burden in the liver and comorbidity of liver cirrhosis or chronic virus hepatitis, the diagnosis and treatment of liver immune-related adverse effects are more difficult. The incidence of immune checkpoint inhibitor (ICI)-related hepatotoxicity is about 2–30% and severe cases are very rare [56]; however, hepatitis accounts for 16–22% of all fatal immune-related adverse events [55]. The accumulation of personal experiences in the management of these cases will be very slow, while collaborations between oncologists and hepatologists may refine the management of ICI-related hepatotoxicity.

## Other emerging targets and agents

Much effort has been made to identify the driver mutation in HCC, but most of the identified somatic mutations were not actionable [57]. All approved targeted drugs for advanced HCC were not specifically developed for HCC. Specific targeting agents for HCC may be not feasible in the near future, but there are some promising molecular targets in drug development for HCC.

### Colony-stimulating factor-1/CSF-1 receptor

Macrophages play a critical role in the progression of HCC, and colony-stimulating factor-1 (CSF-1) is the major chemokine for the recruitment of macrophages [58]. A preclinical study found that PLX3397, a CSF-1 receptor (CSF-1R) inhibitor, showed robust anti-tumor effects in xenograft HCC models [59], and the effects of sorafenib were enhanced when combined with macrophage-depleting drugs [60]. Several agents targeting CSF-1/CSF-1R axis (e.g., PLX3397, JNJ-40346527, and BLZ945) are currently being investigated in clinical trials for solid tumors including HCC.

#### CD47

CD47 is expressed on cancer cells, which can bind to SIRPα on macrophages and serve as a “do not eat me” signal usually presented by normal blood cells; it enables cancer cells to evade immunosurveillance by macrophages or other phagocytes [61]. When administered to patients with lymphoma together with rituximab, 5F9, which occupies the CD47 receptor, showed promising anti-tumor efficacy in a phase Ib study [62]. Preclinical studies also found that CD47 blockage inhibited tumor growth [63] and showed synergic effects with sorafenib [64] in HCC mouse models.

#### Other immunotherapies

CTLA-4 is another extensively studied co-inhibitory receptor. CTLA-4 is a CD28 (T cell co-stimulatory protein) homolog and outcompetes CD28 binding affinity for B7 on antigen-presenting cells. CTLA-4 is also found constitutively expressed in regulatory T cells. Ipilimumab, an anti-CTLA-4 antibody, was approved as monotherapy for melanoma and in combination with nivolumab for renal cell carcinoma by USFDA. In the CheckMate 040 study, the combinational use of ipilimumab and nivolumab was also studied in sorafenib-treated patients with advanced HCC [65]. A total of 148 patients were randomized to three arms with different dosages of ipilimumab and nivolumab. Overall, the combination showed a more potent anti-tumor effect than nivolumab monotherapy with a higher ORR (31% vs 14%) [48, 65], the median DOR was 17 months, and the 24-month OS rate was 40%. Although the combination was well tolerated, the rate of grades 3–4 treatment-related adverse events were also much higher than nivolumab monotherapy (37% vs 18%).

Besides anti-PD-L1/PD-1 antibodies and anti-CTLA-4 antibodies which have already shown clinical efficacy and had led to FDA approval in the treatment of various solid tumors including HCC [66], other co-inhibitory receptors, such as Lag-3, T cell immunoglobulin mucin-3 (Tim-3), and TIGHT were promising targets to be translated to the clinical development [67]. Preclinical studies established the anti-tumor effects of targeting Tim-3 as monotherapy or in combination with other agents in various types of malignancies (summarized in Ref. [68]). Patients with advanced HCC will also benefit from the clinical development of the next generation of ICIs targeting Tim-3, Lag-3, and TIGHT in solid tumors [69].

#### Fibroblast growth factor receptor 4

FGF19 was identified as an oncogenic driver via its receptor, fibroblast growth factor receptor 4 (FGFR4). The aberrantly activated FGF19/FGFR4 signaling pathway was identified as driving hepatocarcinogenesis [70] and was associated with poor prognosis in patients with HCC [71]. BLU-554 is a potent and highly selective FGFR4 inhibitor. In a phase I study of BLU-554 in HCC patients, the ORR was 26% (5/19, including 1 CR and 4 PR) in the subgroup with high FGF19 expression, accounting for 27% of the study participants [72]. FGF401, another FGFR4 inhibitor, was investigated as a monotherapy or in combination with PDR001 in HCC patients with positive FGFR4 and KLB (a FGF19 co-receptor) expression (NCT02325739).

#### CD105

A previous study found that CD105 (endoglin)-positive HCC endothelial cells showed increased apoptosis resistance, motility, and proangiogenic properties. These cells acquired more resistance to adriamycin, 5-fluorouracil, and sorafenib than their counterparts without CD105 expression in normal liver tissue [73]. The combination of TRC105 (an anti-endoglin antibody) and sorafenib demonstrated encouraging evidence of efficacy, including a 25% partial response rate and a durable PR in HCC patients with measurable disease in an early stage clinical trial [74, 75].

Other small molecular agents, donafenib (kinase inhibitor of Raf and VEGFRs) (NCT02645981) and apatinib (kinase inhibitor of VEGFR2) (NCT02329860), have been investigated in phase III studies. Both studies were closed, and the results will be released shortly.

## Novel approaches to improve the effect of systemic treatments

Two approaches may improve treatment efficacy using currently approved agents. The first strategy is to enrich patients with biomarkers. Several biomarkers have been found to be associated with sorafenib efficacy [76], but none of them were prospectively validated. The only proved biomarker is AFP for ramucirumab treatment. Although some studies showed PD-L1 expression on tumor tissue and tumor mutation burden was associated with the effect of PD-L1/PD-1 antibody treatment [77], there is no biomarker approved for predicting the efficacy of ICI in HCC [47, 54].

The second approach is the combination of therapies targeting various pathways.

### Combination therapy of anti-angiogenesis and PD-L1/PD-1 antibodies

Anti-angiogenic drugs targeting the VEGF-VEGFRs signaling pathway are the first-line and second-line therapies approved for HCC. In all phase III studies that led to the approval of molecular targeting therapies, the mOS for patients with advanced or unresectable HCC was about 1 year [11, 12, 20], and there may be a ceiling of effects for these TKIs [78]. However, all combinational therapies with sorafenib, including systemic chemotherapy (doxorubicin) [79], hepatic arterial infusion chemotherapy [27], tigatuzumab (a death receptor-5 agonist) [80], erlotinib (an EGFR inhibitor) [24], and TACE [25], have failed to improve mOS compared with sorafenib monotherapy.

ICIs may be promising for combination therapy with sorafenib and other anti-angiogenic drugs because the major toxicity profiles of TKIs and ICIs are not overlapped. Early stage clinical studies in HCC and late-stage studies in other solid tumors have shown that the toxicity of these two categories’ combination is manageable (Table 1).

**Table 1:** Safety and efficacy of combination treatment in patients with advanced HCC

Combinations	Number of patients	ORR (RECIST v1.1)	Median PFS (months)	Grade 3/4 AE	Reference
Apatinib + camrelizumab	16 (second line)	50%	5.8	NA	Xu et al. [54]
Lenvatinib + pembrolizumab	30 (26/30 as first line)	53.3%	9.7	NA	Kudo [81]
Bevacizumab + atezolizumab	68 (first line)	34%	14.9	25%	Pishvaian et al. [82]
Durvalumab + tremelimumab	40 (first and second line)	25%	NA	25%	Kelley et al. [83]
Ipilimumab + nivolumab	148 (second line)	31%	NA	37%	Yau et al. [65]
Axitinib + avelumab	22 (first line)	13.6%	5.5	72.7%	Kudo et al. [84]
FOLFOX4 + camrelizumab	34 (first line)	26.5%	5.5	5.9%	Qin et al. [85]

*ORR* objective response rate, *PFS* progression-free survival, *AE* adverse events, *NA* not available

In a phase Ib study evaluating the safety of lenvatinib in combination with pembrolizumab in 13 evaluable patients with unresectable HCC (NCT03006926) [86], no new adverse event was identified, with a PR rate of 46% (6/13). Another phase I study investigating the combinational use of camrelizumab and apatinib in patients with advanced solid tumors showed manageable toxicity, with a PR of 50% (8/16) in the evaluable HCC patients [54]. The combination of lenvatinib and pembrolizumab showed promising anti-cancer activity in a phase II study in renal cell carcinoma, with the ORR as high as 66.7%, and the mPFS as 17.7 months [87]. The successful experience in renal cell carcinoma has shed light on drug development for HCC, and the combination of TKI and ICI can be anticipated to further improve HCC outcomes based on multiple mechanisms (reviewed in Ref [88]). For example, anti-angiogenesis treatment may increase the efficacy of immunotherapies by targeting angiopoietin 2 and hepatocyte growth factor pathways, while immunotherapies, especially checkpoint inhibitors, may increase the efficacy of anti-angiogenesis treatment, reportedly by eliciting antibody-dependent cytotoxicity on endothelial cells followed by destructing tumor vasculature [88]. The highest ORR was reported in several small trials testing combination treatment of anti-angiogenesis agents with PD-1 antibodies, which are summarized in Table 1. Further evaluation of the safety and efficacy in phase III clinical trials is warranted as a top priority in drug development for advanced HCC by the pharmaceutical industry. The ongoing large phase III clinical trials, which most concerned the combination therapy with anti-angiogenesis and ICI in HCC patients, are listed in Table 2.

**Table 2:** Ongoing phase 3 clinical trials for advanced stage or unresectable hepatocellular carcinoma

Trial	Lines	Arms	Clinicaltrials.gov identifier	Sponsor
IMbrave150	First line	Atezolizumab + bevacizumab vs sorafenib	NCT03434379	Roche
	First line	Tislelizumab vs sorafenib	NCT03412773	BeiGene
ZGDH3	First line	Donafenib vs sorafenib	NCT02645981	Zelgen
HIMALAYA	First line	Durvalumab or durvalumab + tremelimumab vs sorafenib	NCT03298451	AstraZeneca
LEAP-002	First line	Lenvatinib + pembrolizumab vs lenvatinib	NCT03713593	MSD + Eisai
COSMIC-312	First line	Cabozantinib + atezolizumab vs sorafenib vs cabozantinib	NCT03755791	Exelixis
SHR-1210-III-310	First line	Camrelizumab + apatinib vs sorafenib	NCT03764293	Hengrui
ORIENT-32	First line	Sintilimab + IBI305 vs sorafenib	NCT03794440	Innovent
PHOCUS	First line	Pexa-Vec followed by sorafenib vs sorafenib	NCT02562755	SillaJen
KEYNOTE-394	Second line	Pembrolizumab vs placebo in Asian pts.	NCT03062358	MSD
AHELP	Second line	Apatinib vs placebo	NCT02329860	Hengrui

Anti-angiogenic drugs that failed to show efficacy in HCC due to intolerability and consequently insufficient exposure may be rescued by the combination with an ICI. In a phase II study, bevacizumab at 5–10 mg/kg every 2 weeks did show anti-tumor activity in HCC patients with an ORR of 13%, and 65% were progression-free at 6 months [89]. However, serious bleeding occurred in 11% of the HCC patients and held back further phase III studies. However, in more carefully selected HCC patients, when combined with atezolizumab, an anti-PD-L1 antibody, bevacizumab at a dose of 15 mg/kg every 3 weeks showed acceptable tolerability with promising results; ORR was 34% and 6-month PFS was 71% in a phase Ib clinical trial in 68 HCC patients [82]. The combination was further investigated as first-line treatment compared with sorafenib in a phase III study (IMbrave150 study) and the results are to be released at the end of 2019. Tivantinib, a non-anti-angiogenic TKI targeting MET, failed to improve patient OS in a phase III study, probably due to dose-limited toxicity and inadequate dosage [90, 91]. There are ongoing early phase clinical trials evaluating the safety and tolerability of combination therapy of MET inhibitors and ICIs (NCT02795429).

#### Reformative loco-regional therapies

Chemotherapy agents, whether used alone [92] or in combination with sorafenib [79], or in modified formulation [93], failed to show benefits in RCT settings. However, the intratumoral drug concentration enrichment strategy seems to be promising. In a phase I trial [94], 10 patients with primary or secondary liver tumors received a single intravenous infusion of lyso-thermosensitive liposomal doxorubicin, followed by extracorporeal focused ultrasound exposure at a single liver tumor site. This treatment resulted in an average 3.7 times increase of intratumoral doxorubicin concentrations.

Local administration of an oncolytic and immunotherapeutic vaccinia virus JX-594 (Pexa-Vec) showed promising anti-tumor effects in a phase II dose-finding trial [95]. The response rates were 15% (mRECIST criteria) and 62% (Choi criteria). The intrahepatic disease control (50%) was equivalent in injected and distant non-injected tumors. The mOS was 14.1 months and 6.7 months in patients with high and low infused dose, respectively. An ongoing phase III study (PHOCUS study, NCT02562755) is evaluating Pexa-Vec followed by sorafenib vs sorafenib monotherapy in first-line therapy for advanced HCC [96].

## The future of liver cancer treatment

A molecule-based enrichment system to guide targeting therapies in HCC is not yet available. Although the phase III study REACH-2 showed improved survival in the biomarker AFP-enriched population with advanced HCC [45] and led to the approval of ramucirumab for second-line therapy for advanced HCC, AFP was not the molecular target of ramucirumab. There are also no biomarkers guiding patient selection for ICI treatment in advanced HCC. Further efforts to identify enrichment biomarkers are merited.

No agent has been proved effective as an adjuvant therapy for HCC yet. A potent adjuvant therapy for HCC patients with high risk of recurrence is more valuable. The ongoing studies, such as Checkmate-9DX (NCT03383458) and KENOTE-937 (NCT03867084), evaluate the effect of nivolumab or pembrolizumab in adjuvant settings for HCC patients with a high risk of recurrence after resection or ablation. Other ICIs are also being evaluated as adjuvant therapies (Table 3). Adjuvant therapies for Chinese patients are of greater value. According to Chinese guidelines for the diagnosis and treatment of liver cancer [97], indications of liver resection can be expanded to patients at BCLC B stage (Chinese stages IIa and IIb) or partly BCLC C stage (Chinese stage IIIa). These patients are at high risk of disease recurrence, and an effective adjuvant therapy with high efficacy and acceptable toxicity will improve the long-term survival in these patients.

**Table 3:** Ongoing phase 3 clinical trials for intermediate or early stage hepatocellular carcinoma

Trial	Settings	Arms	Clinicaltrials.gov identifier	Sponsor
EMERALD-1	intermediate stage, first line	TACE + durvalumab ± bevacizumab vs TACE + placebo	NCT03778957	AstraZeneca
CheckMate 9DX	Early stage, adjuvant therapy	Nivolumab vs placebo	NCT03383458	BMS
EMERALD-2	Early stage, adjuvant therapy	Durvalumab + bevacizumab vs durvalumab + placebo vs placebo	NCT03847428	AstraZeneca
KEYNOTE-937	Early stage, adjuvant therapy	Pembrolizumab vs placebo	NCT03867084	MSD
JUPITER 04	Early stage, adjuvant therapy	Toripalimab vs placebo	NCT03859128	Junshi

*TACE* transcatheter chemoembolization

Nivolumab, pembrolizumab, and three PD-1 antibodies manufactured in China (toripalimab, sintilimab, and camrelizumab) have been approved by the NMPA in China, but HCC is not an approved indication. Off-label use of anti-cancer drugs is common in China. The price of the three PD-1 antibodies manufactured by local pharmaceutical companies is about one third that of nivolumab or pembrolizumab (less than 2000 US dollars per month). Drug development by local pharmaceuticals will provide Chinese patients with more affordable medications.

As for patients with intermediate stage HCC, all the studies evaluated the combination of sorafenib and TACE failed to show an improved mOS as compared with sorafenib or TACE monotherapy [25, 98, 99]. The ongoing TACTICS study comparing TACE plus sorafenib vs TACE alone in unresectable HCC showed an improved PFS (25.2 vs 13.5 months, *P* = 0.006), but the OS data were immature at the data cutoff [22]. Combining ICI may improve the efficacy of TACE monotherapy based on several potential synergic effects between loco-regional therapies and ICI (reviewed in Ref. [100]). For example, the ongoing EMERALD-1 study (NCT03778957) compares TACE plus durvalumab (an anti-PD-L1 antibody), with or without bevacizumab, with TACE plus placebo. In the near future, the efficacy of TACE may be improved by an ICI; therefore, patients with intermediate HCC may also benefit from systemic therapy.

## Conclusion

The systemic therapy for the patients with advanced HCC will be changed by the novel molecular targeted therapy and immunotherapy. Treatment algorithm for early stage and intermediate stage HCC is also evolving with the emerging agents or novel strategies combined with the existing treatment modalities, all of which may improve patients’ survival in general.

## Data Availability

Not applicable.

## Abbreviations

CR: Complete response
HCC: Hepatocellular carcinoma
ICI: Immune checkpoint inhibitor
ORR: Objective response rate
OS: Overall survival
PD-1: Program death-1
PD-L1: Program death-1 ligand
PFS: Progression-free survival
PR: Partial response
RECIST: Response Evaluation Criteria in Solid Tumors
TACE: Transcatheter chemoembolization
TKI: Tyrosine kinase inhibitor
